# On a Case of Apoplexy of the Spinal Gray Substance, Attended with Convulsions, with Pathological Examinations and Remarks

**Published:** 1865-04

**Authors:** M. Gonzalez Echeverria


### ON A CASE OF APOPLEXY OF THE SPINAL GRAY SUBSTANCE, ATTENDED WITH CONVULSIONS, WITH PATHOLOGICAL EXAMINATIONS AND REMARKS.

BY M. GONZALEZ ECHEVERRIA, M.D.

In the study of pathology two sciences are necessarily involved, and they refer respectively to the living and dead organism: the one of these sciences is physiology, the other morbid anatomy. The one investigates the phenomena of life, and its object is to establish their laws; the other controls or makes the law good by disclosing the relationship between disease and the anatomical elements; for we cannot understand disease without comprehending the alterations or changes in the physical or chemical relations of the organic elements. It frequently occurs, however, that physiological experiments and pathological investigations are incompatible in their results; that the direct observation of the one renders invalid the theories of the other. The discordance is, among other instances, fully illustrated in the unsettled opinion as regards the way in which sensative and motory impressions are conveyed through the different parts of the spinal cord. Assuredly, the reasons of this discordance need not be insisted upon here. Experimental physiology has unquestionably determined great revolutions in modern medicine; but the fact is, that though practical in its scope, it is liable to become quite speculative from the vastness of the issues in the application of experiments to disease, and very frequently, too, from not availing itself of the important assistance which histology brings to the investigation both of normal and morbid phenomena, and, perhaps, still more to the proper performance of the very physiological experiments themselves.

The following case may serve to throw some light on the functions of the gray substance of the spinal cord, and on the symptoms accompanying its alterations or injuries. The examination of the cord I was compelled to make whilst the organ was in a fresh condition, in order to preserve it for presentation to the New York Pathological Society, where it was shown by Dr. L. A. Sayre, in April, 1864. I regret very much that, after the presentation, putrid softening should have prevented a more satisfactory microscopical examination of the structural changes in different sections of the cord, submitted to previous maceration in chromic acid.

P. R., aged 18, of slender but well developed form, enjoyed uninterruptedly good health to January, 1863, when he was attacked with rheumatism, extending to all the joints. His parents were both living: the father had chorea when young, and recovered completely from it. The boy was seized with slight imperceptible twitchings, about the 10th of April, 1864, one week before the day I visited him in consultation with Dr. Sayre. Previous to that date, he had only complained, to use his own language, of "pain in the neck, from too much hanging of the clothes on that part." At this time, Dr. Sayre ascertained that there were pain and spasmodic contractions of the upper limbs, produced upon pressure on the back of the neck. There was no fever, no headache, nor any other manifest symptom. The convulsive movements, gradually increasing, became so violent that he took to his bed, and remained there for three days prior to our visiting him in consultation. He retained his intellect perfectly clear, and had no fever during this period, nor did he complain of pain in his head. When we saw him, he was lying in bed, restrained by straps tied to the limbs, which were thrown into constant violent convulsions. The movements were quite involuntary and instantaneous, rapidly succeeding each other, and of similar character to the convulsions of chorea. He could execute every movement of the limbs; indeed, he could move them suddenly, but if he attempted to perform the movement slowly, the power of co-ordination failing, the convulsions occurred. Pinching and pricking were felt in the skin of the trunk and extremities, and he also complained of pain in the back and in the limbs under the straps, the skin being in those parts already bruised by the rough jerking. There was no enlargement or inflammation of the joints, and he did not feel the limbs cold or numb. He could, with great difficulty, answer the questions during our long examination, evidently showing that the speech was impeded from incapacity of co-ordination in the movements of the muscles of the tongue. There was no trismus. The tongue could be protruded without difficulty, and was moist and clear. Deglutition was impossible from pharyngismus at every attempt to pass anything into the œsophagus. Noise did not seem to exert any influence on the production of the convulsions. The eyes appeared normal, with very slight injection of the conjunctivæ, and no remarkable change in the size, shape, or color of the pupil. Nor did the patient give any signs of photophobia. The countenance was rather pale, with an expression of distress, and the surface of the body was dry and cool, perspiration being only perceptible on the palm of the hands. Respiration was very labored, and restlessness of the patient prevented our ascertaining the rhythm of the pulse, which was nearly made out 21 in about eight minutes, and found very little resisting. The abdomen was contracted and tympanitic, gas escaping from the bowels during the paroxysms; constipation had existed for four days. There was no retention of urine, which was voided shortly before our arrival. The urine was acid, clear, and free from albumen or sugar. Crystals of basic phosphates were observed on microscopical examination. There was no erection of the penis, nor its abnormal development produced by the habit of masturbation. The patient had had no food, nor any rest for nearly seventy hours. He had been cupped on the back of the neck.

Under the above circumstances, it was determined to resort to anæsthesia to arrest the convulsions, and when controled, to sustain the patient by strong beef-tea, stimulants, and coffee, and to act on the bowels by a turpentine and castor oil injection. The inhalation of chloroform, applied by Dr. G. Doyle, immediately repressed the convulsions, but they recurred, as soon as the anæsthetic was suspended, with the former intensity. The anæsthesia was begun at 7 o'clock P.M., and repeated at every threatening of a paroxysm. At 2 o'clock A.M., the chloroform was withdrawn, it being observed that the pulse was extremely weak and fluttering. During the continuation of the anæsthetic, nourishment and stimulants were given to the patient, as also the turpentine injection. At last, when chloroform had to be discontinued, in consequence of the state of the pulse, the convulsions recurred, abating, however, spontaneously, and being followed by a short period of rest, until 6 o'clock A.M., when death took place quietly; the patient being throughout in a clear state of mind, and having had a copious passage of a dark matter shortly before expiring. It was noticed that each succeeding period of rest was longer than the preceding, until death ensued.

*Post mortem* examination was made forty hours after death, the weather being very cold. Drs. Sayre, Doyle, and Winston were present. The subject was lying on the back, and was not emaciated. The *rigor mortis* had already disappeared. The skin on the iliac fossæ had the bluish discoloration of the early stage of putrefaction, over the joints of the limbs, and on the back of the body it was bruised—the epidermis being completely destroyed on the sacral region. Cadaveric congestion was but little marked on the back. The pupils were largely dilated. It was only allowed to examine the cervical portion of the spinal cord, which was laid bare from the occipital bone to the commencement of the dorsal region. On opening the canal the vertebræ exhibited their normal appearance, without any congestion in their osseous tissue, nor morbid growth protruding on the cavity of the canal. The theca vertebralis was firmly adherent to the canal and intensely congested, adhesions being firmer on the upper than on the lower part of the cervical region. The spinal arachnoid was not thickened nor apaque, and adhered to the cord. The spinal fluid was of a red color, and not considerably increased. The size of the cord seemed to be normal in the cervical portion, removed from the lower extremity of the calamus scriptorius to the beginning of the dorsal region. The white was found firmer than the gray substance. The inferior surface of section in the removed portion of the cord showed that the extravasation of blood had been limited in that part to the posterior cornua, and to both tractus intermediolaterales, the anterior cornua appearing scarcely affected. There was a very slight congestion of the white substance, which seemed in nearly a healthy state. At the upper surface of the section, the gray substance was not so much injured, and exhibited a few spots of extravasation, occupying likewise the posterior portions, while the white substance was apparently in a normal condition. On dividing the cord through the posterior median line, apopletic effusions were found along the gray substance, most of them located nearer to the posterior than to the anterior cornua, and a few extending upon the whole width of the gray substance. The capillary vessels were to the naked eye increased around these extravasations, of a bright scarlet, non-coagulated blood. The congestion of the white substance was only marked in some spots near the posterior median fissure. The finger passed through the foramen magnum could detect firm adhesions between the cerebellum and the membranes, and between these and the occipital bone.

On microscopal examination of the white substance, the nerve-fibres, both in the anterior and posterior columns, were found with the cylinder axis in a state of granular disintegration, surrounded by a fine granular amorphous matter, and filaments of connective tissue. The capillary vessels had undergone a granular alteration only in the vicinity of the gray substance, which, indeed, was the part extensively diseased. In this substance most of the capillaries appeared distended or torn, with their coats granular throughout. The varicose condition of the minute vessels was very plain, when magnified to 100 diam. The gray substance was composed of a connective tissue, filled with very fine granular amorphous matter, containing very few fatty globules, and in some places mixed with crystals of hæmatosine: rarely, there were also spherical nuclei, bearing a resemblance to the *myelocites* described by Ch. Robin, in the gray substance of the nervous centres. None of these were found with the white substance, and most of them were in a state of disintegration. In the specimens here referred to, and which were taken from the substance of the posterior cornua, there were, besides a few broken nerve-fibres, two cells, one of them corresponding by its characters to the ganglionic or sympathetic cells, described by Jacubowitsch, and surrounded by a sheath containing several small nuclei. Both cells were very glanular, without any nuclei, and dark from infiltration of pigment granules and of the coloring matter of the blood. Large multipolar cells with similar aspects were found in preparation of the anterior cornua. The conical epitholeum of the central canal had undergone considerable disintegration, in most of the places where the central part of the gray substance had not been encroached upon by the effusion of blood. The alteration in the nerve-fibres and cells in the ganglia of the posterior roots was alike, though in a much less degree, to that of the same elements in the spinal cord. Their capillary vessels were very abundant and enlarged. The vascularity of the membranes was considerable, the vessels being in several places surrounded by lympth infiltrated into the connective tissue. No inflammatory corpuscle was observed in any of the different preparations.

I will not dwell at any length on the share which hereditary and rheumatic influences might have had on the etiology of the case already reported. Neither will I discuss the question whether it should be looked upon as an instance of true chorea. However this may be, it is quite certain, that the apyretic character of the disease, the absence of delirium, hyperæsthesia, and paralysis, with the several other symptoms, reject the idea of considering it as a case of spinal meningitis; although, on examination of the cord, a few signs of the early stage of meningitis were detected, but without the usual increased quantity of cerebro-spinal fluid, or the congestion and inflammation, or softening of the tissue of the cord itself.

Incomplete as it is, the pathological examination, compared with the symptoms exhibited by the case, presents unquestionable value as regards the functions of the two substances of the spinal cord. One fact strikes at once, *i. e.*:—the unimportant derangement of the eye with such an extensive injury of the cilio-spinal region. It is true that the lesion was almost limited to the gray substance of the cord, and I do not wish to pronounce upon any interpretation of the phenomenon, which should seem to reveal that the vaso-motory nerve-fibres must ascend in very small number through the gray substance of the cervical portion of the cord. The case furthermore proves, that irritation of the gray substance of the cord does not always produce hyperæsthesia, nor does alteration in this part of the cord and anæsthesia, or paralysis of movement, always accompany each other, as advanced by Dr. Brown-Séquard.* It again contradicts the opinion of the same skilful physiologist, who, in accordance with the above views, states that the transmission of sensitive impressions chiefly takes place through the central gray matter.† The evidence afforded by this case grows stronger, since it agrees with a similar observation recently made by Lockhart Clarke. This great anatomist, with the ability for which he is so conspicuous, examined the spinal cord in a case of paralysis, attended by Dr. Reynolds, and found the gray matter of the cord almost wholly destroyed on the lower part of the cervical region, without the slightest diminution of sensibility in the trunk or lower extremities, and with painful hyperæsthesia in the muscles of the left arm, and none in the right arm, *although equal portions of the central gray substance were destroyed on the right and left sides.* There was, in addition, incomplete paralysis of motion, limited to the left arm, and due to a large indurated mass, surrounded by softening of the substance in the middle of the right cerebral hemisphere, and to softening also of the fornix, corpus callosum, and of the thalamus opticus in the right side.‡

It might be supposed that anæsthesia in this, like as in other similar cases, was unnoticed, because the remaining nerve-fibres of the gray substance compensated for the loss of sensibility. Such an opinion put forward by Dr. Brown-Séquard to explain the rarity of anæsthesia in cases of deep alterations of the cord, requires the previous demonstration of a sphere of action in the nervous force, capable, like that of electricity, of extending itself over a large surface. Indeed, some physiologists admit the fact as probable in the very extremities of the nerve-fibres, when, free from its oily content, the fibre is reduced to the cylinder axis; but were the hypothesis true, such a nervous atmosphere could never be extensive or intense enough to conceal the existence of some local anæsthesia, for reasons too obvious when we consider the arrangements of the nervous elements. It is true, the experiments of Van Deen and M. Schiff, show that even microscopical fragments of the spinal gray substance are capable of transmitting sensitive impressions, yet, it has been proved by observations, recorded by Cruveilhier, Lockhart Clarke, and Schiff himself, that under such circumstances the sensation is dull and its perception very slow. And Brown-Séquard, him-

* "Lectures on the Central Nervous System," pp. 124, and following.
† Loc. cit., pp. 23 and 130.
‡ Brit. and Foreign Med. Chir. Review, July, 1864, pp. 200, and following.

self, lays it down as a conclusion, that "anæsthesia in cases of disease of the spinal cord, is a symptom indicating that the gray matter is altered. This conclusion seems to hold good, also, with respect to the loss of each of the various kinds of sensibility." Before coming to this conclusion, he states, "that the nerve-fibres employed in the transmission of each of the sensitive impressions of *touch*, *tickling*, *pain*, *heat*, and *cold*, and the peculiar sensation which accompanies muscular contraction are as distinct, one from the other, as they are from the nerve-fibres employed in the transmission of the orders of the will to the muscles."* Assuredly these assertions are not altogether compatible with the preceding theory, by which Brown-Séquard attempts to explain why sensibility is so rarely lost in cases of deep alterations of the spinal cord.

On the other hand, the case wholly confirms the results arrived at by Van der Kolk, who holds that "the gray matter in the spinal cord serves solely for motion, the posterior rather for reflex action and the co-ordination of movements, while sensation is transmitted upwards exclusively through the posterior and lateral medullary columns."†

It is no less important to remark the labored respiration, in connexion with the injury sustained by the tractus intermedio-lateralis in the lower part of the cervical region; tractus which, as demonstrated by Lockhart Clarke, is passed through by the roots of the spinal accessory nerve.

There was, again, a striking similarity between the alteration undergone by the cord, and that resulting from poisoning with strychnine, when, as observed by Van der Kolk and other physiologists, a great congestion and extravasation occur in the gray substance of the cord, with violent convulsions, and neither loss of consciousness nor pain, whilst the medullary or white substance of the cord undergoes very little or no change. Such perfect similarity of cadaveric signs might possibly have awakened the suspicion of this being an instance of poisoning from strychnine. However, the gradual development of the disease, its comparatively long duration, the absence of opisthotonos, acoustic hyperæsthesia, trismus, protrusion of the eyeballs, and of the profuse perspiration attending poisoning from strychnine, as well as the *rigor mortis*, so long persisting in such cases, could not have given ground to that opinion.

Last, though not the least important point. To what extent

* Loc. cit. pp. 125 and 130.

† On the Minute Structure of the Spinal Cord and Medulla Oblongata. New Sydenham Society, London, 1859, p. 77.

did the administration of chloroform favor the congestion and extravasation discovered in the cord? It would, perhaps, be difficult to answer this question, but, it was evident that instead of deriving any permanent relief from the anæsthetic, the spinal cord continued as capable as before its administration of inducing violent convulsive movements, until the exhaustion of its reflex faculty; a fact again disclosed by the liability of epileptics to a renewed attack, when submitted to the influence of chloroform or ether, and decidedly warns against the danger of the indiscriminate use of anæsthetics in the treatment of epileptic diseases.—*N. Y. Med. Journal.*

